# Knowledge and Practice in the Assessment of Frequent Landscape Diseases in Cattle Populations Along the Madhumati River, Gopalganj, Bangladesh

**DOI:** 10.1002/vms3.70622

**Published:** 2025-10-04

**Authors:** Abdullah Al Mamun, Asmita Karki, Hari Prasad Panthi, Nabin Pandey, Esheta Mohtarima, Hoor E Jannat Jaoti

**Affiliations:** ^1^ Department of Animal Science and Veterinary Medicine Bangabandhu Sheikh Mujibur Rahman Science and Technology University Gopalganj Bangladesh

**Keywords:** disease prevention, epidemiology, FMD, husbandry practices, LSD

## Abstract

**Background:**

Cattle have been domesticated by humans for thousands of years for various purposes, including their economic significance. Despite advancements in livestock farming technologies, challenges such as disease outbreaks continue to affect productivity and economic stability.

**Objectives:**

The aim is to investigate the prevalence and factors associated with various diseases in cattle around the Madhumati River in Gopalganj, Bangladesh. The study also offers insights into the current disease landscape, focusing on animal health status, farmer knowledge and treatment and prevention strategies in Gopalganj.

**Method:**

A standardized closed‐ended questionnaire was administered using KoboToolBox (2021.2.4) for data collection. Statistical analyses included descriptive statistics, Fisher's exact test, *χ* ^2^ test, and multivariate logistic regression models with 95% confidence intervals. R (4.4.0) and SPSS (27.0.1) were utilized to explore associations between variables influencing disease prevalence.

**Result:**

The findings of the study indicate that Foot and Mouth Disease (17.39%) and Lumpy Skin Disease (13.04%) were the most prevalent diseases. A linear model (95% CI) predicted that poor husbandry practices, lack of knowledge, and inadequate treatment strategies contributed to disease occurrence in different regions of Gobra, Gopalganj.

**Conclusion:**

This cross‐sectional study investigated the major prevalence rate and causes of diseases in the Gobra region of Gopalganj, identifying FMD and LSD as significant diseases in cattle farming. From the study, we can suggest some strategies to mitigate disease prevalence, including seasonal preventive measures, routine vaccination, deworming protocols, and stringent sanitation practices.

## Graphical abstract

A cross‐sectional study found that Food and Mouth Disease (17.39%) and Lumpy Skin Disease (13.04%) were the most prevalent diseases in livestock, as demonstrated in Gobra, Gopalganj.

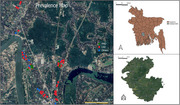

## 1 Introduction

The modern era, especially the post‐industrial revolution, brought significant improvement in the cattle industry. Advancements in breed improvement, integration practices and technological integration make cattle rearing a large global industry (Colominas et al. 2014). Despite the growing demand for the cattle rearing industry, many crucial factors, like outbreaks of diseases, can lead to substantial economic losses and a reduction in productivity. Livestock production remains at peak only if they are free from disease outbreaks. Infectious diseases pose a significant barrier to livestock productivity. Livestock are susceptible to viral as well as bacterial diseases like FMD (Foot and Mouth Disease), Mastitis, Bovine Mastitis, Bovine (Lumpy Skin Disease) and other infectious diseases each year, and account for nearly 20%–25% losses in the world's livestock population (Arzt et al. 2021; Giasuddin et al. 2020).

In Bangladesh, two major diseases, FMD and LSD, share major contributions to economic losses. LSD is imposing a new financial burden on livestock farmers. LSD is responsible for reduced growth rate and productivity as well as increased veterinary cost and animal mortality rate, leading to an economic loss of $80–$120 per infected animal (Biswas et al. 2024; Saqib et al. 2023; Khan et al. 2024).

FMD is caused by an antigenically diverse RNA VIRUS, Foot and Mouth Disease Virus (FMDV). The FMD virus is a member of the genus Apthovirus in the family Picornaviridae. There are seven serotypes of FMD virus: O, A, C, SAT 1, SAT 2, SAT 3, and Asia 1 (Biswas et al. 2024; Khan et al. 2024; Saqib et al. 2023). It possesses rapid transmission from acutely infected animals to healthy animals through respiratory aerosols and droplets or indirectly via environmental mechanisms (Sprygin et al. 2019). The susceptible animals, like cows, buffalo, sheep and goat, show signs of fever, lameness, vesicular lesions and blisters in mouths, tongues, feet and teats. The disease may cause severe reproduction, lactation, growth and draught power losses. Annual financial losses due to FMD in Bangladesh would be 2220.82 million BDT according to the data collected from July 2017 to June 2018 (Giasuddin et al. 2019).

The lumpy skin disease virus (LSDV) is a member of the *Capripoxvirus* genus in the Poxviridae family. LSD possesses different serotypes (Neethling and bovine) according to their virulence and geographical distribution. The symptoms of LSD include fever, swollen lymph nodes and skin nodules. The disease spreads by direct contact with diseased animals, contaminated feed and water, and vectors like flies and mosquitoes (Khan et al. 2024). Susceptible animals, such as cows and buffaloes, display signs like fever, lameness, skin nodules and lymph node swelling. Due to decreased milk supply, weight loss, infertility and increased mortality and morbidity rates, LSD can result in significant financial losses (Sprygin et al. 2019; Tuppurainen et al. 2021). Lumpy skin disease causes high morbidity (26%) and low‐moderate mortality (0%–20%) (Biswas et al. 2020). Vaccination and vector control are two important preventative and control strategies that are essential for reducing the negative effects of LSD (Yuan et al. 2024). The total estimated annual loss due to lumpy skin disease in Bangladesh was 91.33 million US$ found in the Mymensingh region (Chouhan et al. 2022). Recently, the Bangladesh Livestock Research Institute (BLRI, Savar) has developed a live‐attenuated LSD vaccine from local strains (Samad et al. 2025).

In this study, the main focus is on these two contagious viral infections, FMD and LSD. Systemic information was collected about the prevalence of FMD, LSD and other major diseases occurring in the village of Gopalganj area alongside the Madhumati River. The study involves the investigation of the prevalence rate of FMD and LSD along with other important diseases that the farm animals are more susceptible to, the risk factors, farm practices, adoption of treatment facilities and prevention methods, feeding nature and government approaches in controlling these diseases (Burrows et al. 2025; Morgan Bustamante et al. 2024).

The findings and results from the study provide a comprehensive overview of FMD, LSD and other diseases with their epidemiological trends, the present condition of animals, knowledge of farm owners about disease prevalence and infection, and their strategy of treatment and prevention.

## 2 Methods

### 2.1 Study Design and Population

**FIGURE 1:**
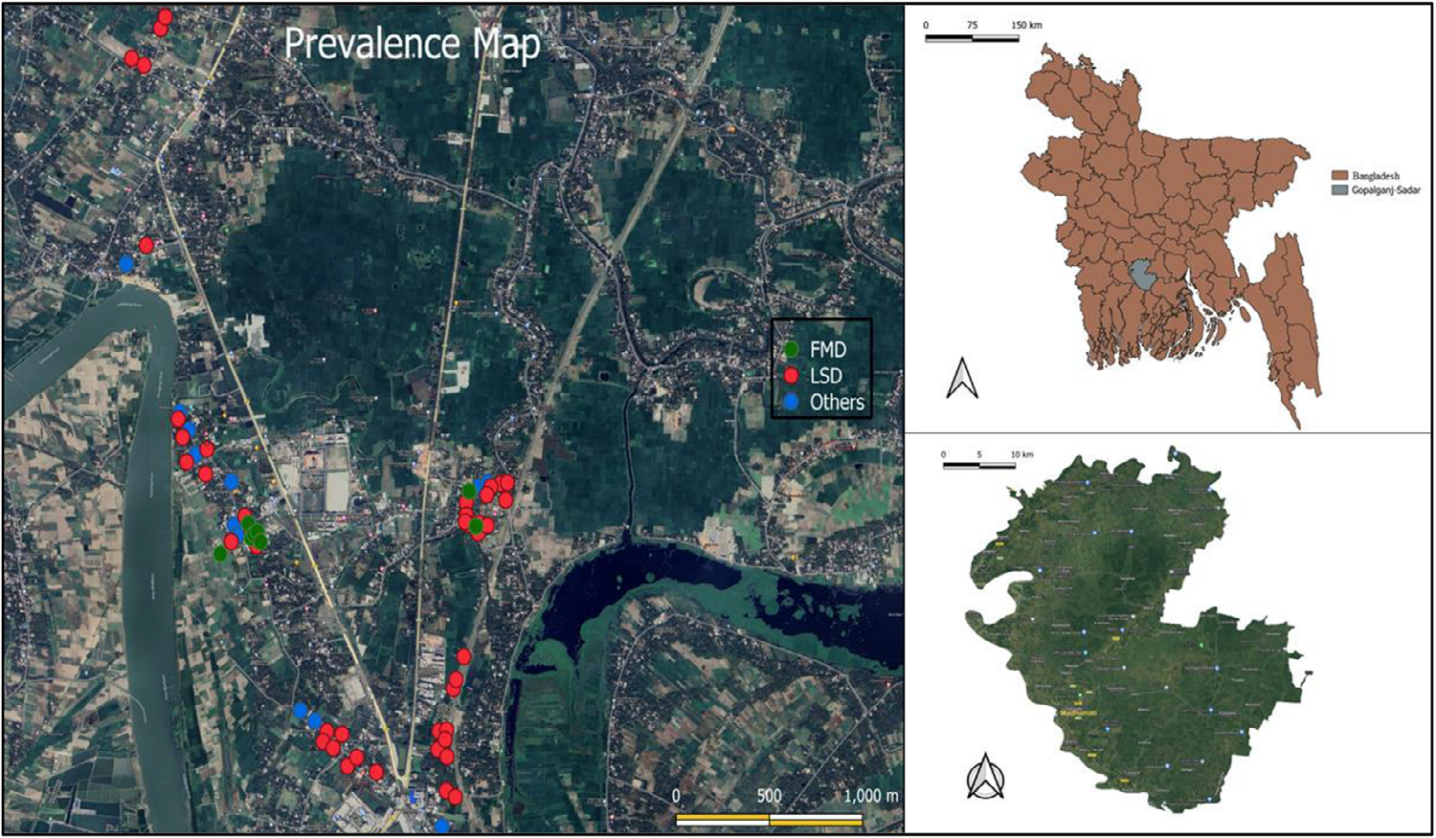
Prevalence rate of diseases.

The study was conducted over 5 months (January to May 2024) in Gobra, Gopalganj Sadar, Gopalganj, Dhaka, Bangladesh, during the peak time of the disease outbreak. Two regions selected: The roadside and the close side of the Madhumati River. The area was selected based on information from the Upazila Veterinary Hospital, Gopalganj, and by local people. Each farm was considered an individual observation in this study. On average, 283 cattle were inspected directly. The QGIS software is used to generate geospatial maps and spatial analytics of the study area (Figure 1).

### 2.2 Collection and Preservation of Data

A standard, closed‐ended questionnaire was used to find the most frequent diseases and associated factors. KoboToolBox software (version 2021.2.4) and the app were used to manage and collect data. There were five subsections of the questionnaire form: Demographic information (e.g. breed, age, sex), husbandry practices (e.g., grazing, feeding, water supply), medication (e.g., type of antibiotics, prebiotics, nutritional supplements), biosecurity (e.g., vaccination, disinfection, routine cleaning, fencing, ventilation) clinical information (e.g., signs arising during a disease condition, any abnormalities) and seasonal effects. The questionnaire data were obtained by visual inspection, reviewing the records and inquiring about the farmer. Questionnaires were provided as additional supplementary files.

### 2.3 Statistical Analysis

Descriptive statistics were used to evaluate the characteristics of the study population. Statistically significant tests like Fisher's exact test and the *χ* ^2^ test were performed to fabricate cross‐tabulations between different observations. Multivariate logistic regression models and 95% confidence intervals (95% CIs) were used to investigate the associations between independent variables influencing the dependent variable in the model. All statistical tests, including risk factors, were considered statistically significant with a *p*‐value < 0.05. R (version 4.4.0) programming language and SPSS (Statistical Package for the Social Sciences version 27.0.1) were used for data analysis, summarization, visualisation, mapping and generating interactive tables.

## 3 Results

Cattle husbandry practices are influenced by geographical location, education and gender. A significant difference was found in the farm location (*p* = 0.044), where 46% of male owners and 23% of female owners had farms close to the river. A higher percentage of female‐owned farms (77%) are far from the river. Educational levels show no significant difference between genders (*p* = 0.8). Most farm owners have only a primary education (65% of females and 71% of males), with very few reaching graduate level (0% of females and 2.9% of males). Around 16% of male owners have received professional training compared to 7.7% of female owners (Table 1).

**TABLE 1:** Demographic information.

Characteristic	Female, *N* = 26*^1^*	Male, *N* = 70*^1^*	*p*‐value*^2^*
**Location of the Farm**			**0.044**
Close to river	6 (23%)	32 (46%)	
Far from river	20 (77%)	38 (54%)	
**Area of Residence**			0.2
Balurmath	5 (19%)	7 (10%)	
Charghata	2 (7.7%)	6 (8.6%)	
Charsunakur	2 (7.7%)	1 (1.4%)	
Ghonapara	3 (12%)	8 (11%)	
GobraMadrasa	2 (7.7%)	2 (2.9%)	
Nilamath	0 (0%)	4 (5.7%)	
Pathalia	9 (35%)	22 (31%)	
ShobhanSarak	0 (0%)	12 (17%)	
Shuterkul	0 (0%)	1 (1.4%)	
South gobra	3 (12%)	7 (10%)	
**Level of Education**			0.8
Graduate	0 (0%)	2 (2.9%)	
High School	7 (27%)	12 (17%)	
Intermediate	2 (7.7%)	5 (7.1%)	
Primary	17 (65%)	50 (71%)	
Professional Training	2 (7.7%)	11 (16%)	0.5

Water sources show significant differences (*p* = 0.001), with tube‐well the predominant source far from the river (57%) compared to close to the river (45%). Feeding practices do not show significant differences (*p* = 0.5), with local markets and grazing common in both locations. “Excellent” health status was reported in 5.3% of total animals in areas where farms are far from the river. Animals are reported as being in good health in both locations (Table 2).

**TABLE 2:** Source of feed and health status.

Characteristic	Close to river, *N* = 38*^1^*	Far from river, *N* = 58*^1^*	*p*‐value*^2^*
**Sources of water**			**0.001**
Other Sources	1 (2.6%)	2 (3.4%)	
Pond	1 (2.6%)	11 (19%)	
Pond and Other Sources	0 (0%)	1 (1.7%)	
Pond and Tubewell	0 (0%)	3 (5.2%)	
River	9 (24%)	2 (3.4%)	
River and Tubewell	9 (24%)	6 (10%)	
Tubewell	17 (45%)	33 (57%)	
**Type of feed and source**			0.5
Grazing, Household	12 (32%)	13 (22%)	
Local Market, Grazing	9 (24%)	23 (40%)	
Local Market, Grazing and Household	12 (32%)	18 (31%)	
Local Market, Grazing, Household and Imported	2 (5.4%)	2 (3.4%)	
Local Market, Grazing and Others	0 (0%)	1 (1.7%)	
Local Market and Household	2 (5.4%)	1 (1.7%)	
Unknown	1	0	
**Current health status**			0.8
Excellent	1 (2.6%)	3 (5.3%)	
Fair	5 (13%)	5 (8.8%)	
Good	29 (76%)	45 (79%)	
Poor	3 (7.9%)	4 (7.0%)	

A significant difference exists in the use of medications between those who self‐medicate without veterinarian consultation and those who use medicines with veterinarian advice (*p* = 0.008). The types of medications used showed statistically insignificant differences (*p* = 0.3), though antibiotics combined with pain relievers are common. Post‐treatment monitoring is reported to be higher in those who have taken veterinarian consultancy (15%) compared to self‐medication (3.2%), which is insignificant (*p* = 0.14). Nutritional supplements are similarly used by both groups, with no significant difference (*p* = 0.2). All respondents reported no side effects from medication. The study revealed that owners who sought veterinary consultancy showed significantly (*p* = 0.015) more regular vaccination and de‐worming practices (47%) compared to self‐medicated owners (19%). This strongly suggests that professional veterinary service is linked to better preventative health measures (Table 3).

**TABLE 3:** Medication and nutrient supplementation.

Characteristic	Self medication, *N* = 31*^1^*	Veterinarian, *N* = 52*^1^*	*p*‐value*^2^*
**Medications to treat animals**	17 (55%)	41 (82%)	**0.008**
Types of medications			0.3
Antibiotics, antihistamines	1 (3.8%)	4 (8.5%)	
Antibiotics, antihistamines	0 (0%)	2 (4.3%)	
Antibiotics, antihistamines and pain relievers	17 (65%)	19 (40%)	
Antihistamines, pain relievers		4 (8.5%)	
Antibiotics, antihistamines		3 (6.4%)	
Antibiotics, pain relievers		1 (2.1%)	
Antihistamine	1 (3.8%)	0 (0%)	
Others	7 (27%)	12 (26%)	
Pain relievers	0 (0%)	1 (2.1%)	
Recurrence	1 (3.2%)	8 (15%)	0.14
**Nutritional supplements**			0.2
Minerals	1 (3.6%)	0 (0%)	
No	17 (61%)	31 (63%)	
Others	1 (3.6%)	0 (0%)	
Vitamin	0 (0%)	3 (6.1%)	
Vitamin, minerals	5 (18%)	12 (24%)	
Vitamin, minerals	4 (14%)	3 (6.1%)	
Side effects of medication			
No	31 (100%)	52 (100%)	
**Vaccination and de‐worming**			**0.015**
Irregular	14 (45%)	18 (35%)	
Never	11 (35%)	7 (14%)	
Regular	6 (19%)	24 (47%)	
Regular, Irregular	0 (0%)	2 (3.9%)	

Farm conditions do not show significant differences (>0.9), with fair conditions being the most common. Animal mortality rates showed no significant seasonal variation and remained consistent across seasons (*p* = 0.7) (Table 4).

**TABLE 4:** Disease incidence across different seasons.

Characteristic	Rainy, *N* = 6*^1^*	Summer, *N* = 47*^1^*	Winter, *N* = 14*^1^*	*p*‐value*^2^*
**Location of the Farm**				**0.007**
Close to river	2 (33%)	22 (47%)	1 (7.1%)	
Far from river	4 (67%)	25 (53%)	13 (93%)	
**Farm Condition**				>0.9
Excellent	0 (0%)	2 (4.3%)	0 (0%)	
Fair	2 (33%)	22 (48%)	7 (50%)	
Good	3 (50%)	16 (35%)	5 (36%)	
Poor	1 (17%)	6 (13%)	2 (14%)	
**Disease occurrences**				0.7
No	1 (17%)	5 (11%)	4 (31%)	
Yes	5 (83%)	38 (86%)	9 (69%)	
**Confirmatory Disease**				0.091
FMD	2 (50%)	4 (9.1%)	1 (8.3%)	
LSD	2 (50%)	33 (75%)	6 (50%)	
Others	0 (0%)	7 (16%)	5 (42%)	
Unknown	2	3	2	
**Mortality**				0.7
FMD	0 (0%)	2 (17%)	1 (20%)	
FMD LSD	0 (0%)	1 (8.3%)	0 (0%)	
LSD	0 (0%)	1 (8.3%)	1 (20%)	
Others	1 (100%)	8 (67%)	3 (60%)	

## 4 Discussion

In this research, data have been collected from multiple areas within Gobra Union in Gopalganj Sadar, Gopalganj. The area included Balurmath, Charpathalia, north and south of Ghonapara and Nilamath. The study identified FMD and LSD as the most frequently occurring diseases in the study population. In addition, other significant disease conditions, including haemorrhagic septicemia, mastitis, tympany, dermatitis, protozoan infections and recurrent seasonal diarrhoea, were also observed in the study area. The environment plays a crucial role in the outbreak of diseases. In this study region, FMD outbreaks predominantly peak during the winter season and post‐monsoon when there is high humidity and low temperature in the environment. Some previous studies also indicated that FMD outbreaks are prominent in the cooler months of the year (Guerrini et al. 2019; Hossain et al. 2023; Hossain et al. 2023).

LSD outbreaks are noticed to be prominent in the summer season by clinical signs like high fever, weakness, anorexia and subcutaneous nodules all over the skin, and acute cases exhibit rupture of nodules and formation of lesions. Similar findings were also found in other studies (Alexandersen et al. 2003). Whereas, Haemorrhagic septicemia (HS) cases peak during monsoon periods when there is high humidity and low temperature in the environment, which provides favourable conditions for the outbreak of diseases. HS is distinguished from major concern diseases, i.e., FMD and LSD, by the observation of clinical signs like high fever, dyspnea, accompanied by frothing at the mouth or nostrils, and edematous swellings in the submandibular region (CABI. 2019). In some cases, swelling spreads to the neck and brisket and sometimes to the forelegs (CABI 2019).

Along with LSD, FMD and other health issues stimulate adverse effects upon animal husbandry. Suboptimal management practices by farmers lead to health complications like mastitis, tympany and diarrhoea. These health challenges compromise animal production and hamper reproduction. This study shows farmers couldn't practice scientific management practices due to various obstructions such as poverty, lack of education, proper training facilities and insufficient coordination from the governmental authority side (Renault et al. 2021). In contrast, few households have managed their farms very well, and the condition of the animals was also far better. As per observation, the main reason behind this is the sound financial condition of the owner, higher education level and training regarding animal farming, at least one of the family members. Previous study indicates that a farmer's education level, financial status, and training level are closely interlinked with each other, with demographic characteristics that combined can create a remarkable impact on the overall management and well‐being of farm animals (Can and Altuğ 2014; Water quality for agriculture 2024). Managemental issues like drinking water and supplied feed are also pivotal in healthy and profitable animal farming. In our study area, close to the Madhumati River, farmers frequently visit the river and take their cattle for bathing and prominently use river water for cooking and drinking purposes for livestock (Landefeld and Bettinger 2003). River water has the potential to harbour a range of disease‐causing pathogens, posing a risk of outbreaks in the community (Magana‐Arachchi and Wanigatunge 2020). Whereas, in the areas far from the river, farmers are bound to use canal or pond water because the underground water has very high levels of iron and arsenic, which are toxic substances for both humans and animals. Farmers generally use household surplus or locally available, cheaper feed that has low nutrient content. Hence, as a result of ragged financial condition, inadequate knowledge level and unfavourable geographical location, farmers are unable to supply clean, fresh drinking water and nutritious feed to their cattle (Hossain et al. 2021). Studies have demonstrated that cattle reared by using deep tube‐well water have far better medication receptivity and fewer occurrences of contagious disease than river water users (Islam et al. 2019; Wu et al. 2011).

Previous studies have shown that biosecurity plays a crucial role in sustainable and profitable animal farming (Msimang et al. 2022; Renault et al. 2021). In this study area, farmers face significant challenges in managing proper biosecurity. Issues such as insufficient space lead to overcrowding, making cleaning and disinfection difficult, resulting in wet and slippery floors that cause injuries and infections by disrupting biosecurity. Scientific ventilation is also rare, causing the accumulation of poisonous gases inside the farms. Despite these challenges, a few farms maintain a fair level of biosecurity with proper cooling and heating facilities, mosquito repellents, and dry, clean floors (Makita et al. 2020).

## 5 Conclusion

The study was conducted to investigate the most prevalent diseases and the underlying factors contributing to their occurrence, which resulted in indicating FMD and LSD as major disease risks for the animals of the Gopalganj region. FMD was found to occur mostly during cooler months, whereas LSD occurs in warm months of the year. Besides diseases like mastitis and HS, other health issues were also seen as potential problems in the village. Timely prevention according to different seasons, regular vaccination and de‐worming, cleaning, disinfecting the herd area at a scheduled time and avoiding river and pond water during the outbreak season might help to reduce the prevalence rate. The study was limited by its reliance on self‐reported data by the locals, which may be subject to bias, and no laboratory tests were performed rigorously. This study can serve as a baseline for researchers and policymakers to assist in understanding disease outbreaks.

## Author Contributions

**Abdullah Al Mamun**: conceptualization, investigation, supervision, data curation, methodology, software, result interpretation, writing – original draft, writing – review and editing. **Asmita Karki**: formal analysis, result interpretation, writing – original draft, writing – review, and editing. **Harry Prasad Panthi**: investigation, formal analysis, interpretation of results, writing – original draft, writing – review, and editing. **Navin Pandey**: conceptualization, writing – original draft, writing – review and editing. **Eshita Mohtarima**: writing – original draft, writing – review and editing, **Hoor E. Jannat Jaoti** – supervision, writing – review and editing.

## Conflicts of Interest

The authors declare no conflicts of interest.

## Ethics Statement

Written informed consent was obtained from the patient and family members. This study was conducted by the Declaration of Helsinki (revised in 2013) and approved by the Ethics Committee of Bangabandhu Sheikh Mujibur Rahman Science and Technology University, Department of Animal Science and Veterinary Medicine.

## Peer Review

The peer review history for this article is available at https://publons.com/publon/10.1002/vms3.70622.

## Data Availability

The datasets used in the study are available from the corresponding author upon reasonable request.
